# Structural Alterations in Cortical Thickness and Volume in Attention Deficit Hyperactivity Disorder (ADHD): An Exploratory Surface-Based Morphometric Study

**DOI:** 10.7759/cureus.106581

**Published:** 2026-04-07

**Authors:** Amanda Kettman, Mario Murakami

**Affiliations:** 1 Research, Alabama College of Osteopathic Medicine, Dothan, USA; 2 Psychiatry, Massachusetts General Hospital, Harvard Medical School, Boston, USA

**Keywords:** adhd, cerebral cortex, fmri, neuroimaging, surface-based morphometry

## Abstract

Purpose: This study aims to investigate the significant clusters of structural alterations across cortical regions associated with attention deficit hyperactivity disorder (ADHD) and explore their potential functional implications.

Methods: A cross-sectional, exploratory surface-based morphometric analysis of cortical thickness and volume in the left and right hemispheres was conducted using FreeSurfer (Martinos Center for Biomedical Imaging) on a publicly available structural magnetic resonance imaging (MRI) dataset. The final analytic sample consisted of 34 observations, including 11 participants with ADHD and 23 participants without ADHD (controls). Participants spanned a range of ages, and group comparisons were performed between ADHD and control participants. Age was included as a continuous covariate in the statistical model to account for developmental variability. Surface-based morphometric data were analyzed with a minimum z-score threshold of 1.3, with cluster-wise correction (cluster-wise p-value (CWP) ≤ 0.05) applied to account for multiple comparisons. Clusters showing significant differences were mapped to anatomical regions, and their functional relevance was interpreted.

Results: Reduced cortical thickness was observed in the right superior frontal gyrus and increased in the left postcentral gyrus. Both hemispheres demonstrated multiple clusters showing significant (CWP ≤ 0.0002) increases or decreases in volume. Increased volume was observed in the right precentral, postcentral, lingual, and fusiform gyri, as well as the left postcentral gyrus, lateral occipital gyrus, inferior parietal cortex, and rostral middle frontal cortex. A decreased volume was observed in the right superior frontal and left supramarginal gyri. Bidirectional volume changes were also noted in the right lateral occipital gyrus and rostral middle frontal cortex.

Conclusion: These findings suggest that ADHD is associated with region-specific alterations in cortical thickness and volume, particularly within executive, motor, and sensory processing regions. Given the mixed-age ADHD cohort and exploratory design, these results should be interpreted as hypothesis-generating, warranting validation in larger, age-stratified cohorts.

## Introduction

Attention deficit hyperactivity disorder (ADHD) is a neuropsychiatric disorder characterized primarily by symptoms of inattention, impulsivity, and hyperactivity [1]. Although traditionally considered a childhood-onset disorder, ADHD frequently continues into adolescence and adulthood, often accompanied by comorbid psychiatric disorders such as anxiety, mood, and substance abuse disorders [2]. These symptoms can interfere with academic achievement, social functioning, and overall quality of life [3,4]. Understanding the neurobiological basis of ADHD is critical, not only for improving diagnostic accuracy but also for developing more effective, targeted interventions. Neuroimaging techniques like magnetic resonance imaging (MRI) allow researchers to examine the brain’s structure and function without surgical intervention, offering insights into the biological mechanisms that may underlie behavioral symptoms.

Structural brain differences have been consistently observed in individuals with ADHD, particularly in regions involved in executive function, emotion regulation, motor control, and reward processing, including the basal ganglia (putamen, caudate, and globus pallidus), amygdala, hippocampus, and anterior cingulate cortex [5-8]. Recent meta-analytic and large-scale neuroimaging studies have expanded these findings to cortical regions, particularly the prefrontal and parietal lobes. Convergent evidence from meta-analyses, mega-analyses, and multimodal MRI approaches demonstrates reductions in cortical thickness, surface area, and gray matter volume in the dorsolateral, inferior, and superior frontal gyri, as well as in parietal regions, alongside disrupted fronto-parietal and fronto-striatal connectivity [9-14]. These alterations appear most pronounced in children and tend to normalize or become less prominent in adulthood, consistent with longitudinal findings or delayed cortical maturation across prefrontal and parietal regions [5,8,10]. Functional MRI and graph-theory analysis further reveal atypical organization and reduced efficiency within the fronto-parietal and cingulo-opercular networks that support attention, working memory, and executive control [15,16]. Collectively, these findings underscore the central role of fronto-parietal circuitry in ADHD and emphasize the need for precise surface-based methods to further characterize cortical alterations.

While numerous neuroimaging studies have examined cortical volume and thickness in individuals with ADHD, many relied on voxel-based or region-of-interest (ROI)-based methods [17]. Surface-based morphometry (SBM) offers more precise vertex-wise measurement across the cortical surface but remains underutilized in ADHD research. Furthermore, few studies have applied strong statistical correction methods to ensure the reliability of SBM findings. This gap highlights the need for comprehensive SBM-based investigations to better characterize ADHD-specific structural brain differences.

To address this gap, we applied SBM to explore cortical volume and thickness variations between individuals with ADHD and neurotypical controls. Our objective was to characterize patterns of cortical structural variation and to interpret these exploratory findings in the context of existing literature on ADHD-related neurobiology, while acknowledging that demographic differences between groups may limit the specificity of findings. However, publicly available neuroimaging datasets often include developmentally heterogeneous samples, which may limit the feasibility of strict age-matched comparisons and necessitate alternative analytical approaches, such as covariate-based adjustment. Given the nature of the dataset and study design, this analysis is intended to be exploratory and descriptive, rather than to establish causal or functional relationships.

## Materials and methods

Study design**

This cross-sectional comparative study uses SBM to explore volume and cortical thickness variations between individuals with ADHD and healthy controls. A quantitative approach was used to analyze brain structure using FreeSurfer (Martinos Center for Biomedical Imaging, Charlestown, MA, USA).

Dataset

Structural T1-weighted whole-brain MRI was used in this study, sourced from the publicly available OpenNeuro.org repository, specifically the dataset titled “Response inhibition and selective attention in adults and children with and without ADHD” (DOI:10.18112/openneuro.ds003500.v 1.2.0) [18]. The dataset includes functional and structural MRI data for a total of 38 participants. Four subjects were excluded from the analysis. One was due to a variation in the T1 structural imaging parameter, and another was due to a variation in the voxel size and aspect ratio. The remaining two were due to errors in the preprocessing step. The final analytic sample consisted of 34 subjects (11 ADHD and 23 controls), including 15 males and 19 females. Within the ADHD group, 7 were male and 4 were female, and within the control group, 8 were male and 15 were female. The scans were defaced using the pydeface tool (Poldrack Lab, Stanford University, Stanford, California) in order to de-identify structural images according to the HIPAA Privacy Rule.

Variables

Cortical thickness, measured as the distance between the pial and white matter surfaces, and gray matter volume were analyzed using surface-based morphometry with FreeSurfer.

Imaging acquisition

T1-weighted structural MRI scans were acquired using a 1.5 Tesla General Electric (GE) Signa Excite scanner, quadrature birdcage head coil utilizing Spoiled Gradient Recalled (SPGR) sequence [18]. T1-weighted SPGR images were collected using the following parameters: TR = 21 ms, TE = 8 ms, matrix size = 256 × 256, slice thickness = 1 mm, number of slices = 124, voxel size = 0.86 x 0.86 × 1 mm, and flip angle = 20°.

Imaging processing

**Figure 1:**
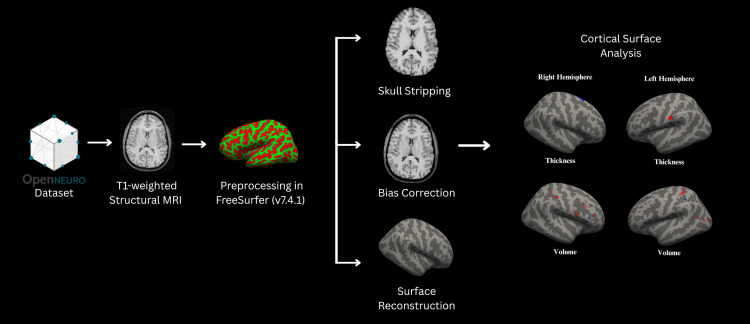
Surface-based morphometry workflow

T1 brain images were analyzed using FreeSurfer 7.4.1 (http://surfer.nmr.mgh.harvard.edu/) command recon-all [19]. Cortical thickness was measured as the distance between the pial (outer boundary of the gray matter) and white matter (inner boundary where the gray matter meets the white matter) surface. Surface-based morphometry was employed to analyze the total volume of gray matter structures. The imaging processing followed the standard Freesurfer pipeline, including skull stripping (removing non-brain tissue) and alignment to Montreal Neurological Institute (MNI) space, as seen in Figure 1.

Bias

Data were sourced from an open-access repository with established protocols to ensure consistent recruitment and inclusion/exclusion criteria. A cluster-wise correction (cluster-wise probability (CWP) ≤ 0.05) was utilized in the statistical analysis to increase specificity and reduce the likelihood of false positives. Differences in imaging parameters (T1 structural imaging, voxel size, etc.) were identified, and the subjects were excluded from the analysis to prevent confounding effects and ensure homogeneity of the dataset. The dataset included adult and pediatric subjects, and the estimated total intracranial volume and age were used as variables in the statistical model in order to decrease variability.

Statistical methods

For the whole-brain analysis, group was included as a discrete factor, with age and estimated total intracranial volume as covariates. Cortical clusters were smoothed at 10 mm full width at half-maximum (FWHM) as recommended per standard settings. Statistical analyses were performed using FreeSurfer’s mri_glmfit tool (FreeSurfer version 7.4.1), which applies a general linear model (GLM) at each vertex of the cortical surface. Left and right hemispheres were analyzed independently, following standard FreeSurfer procedures. For group comparisons, vertex-wise t-tests were performed, and t-statistics were converted into Z-scores. 

Z-values were used as the test statistics and are reported in the tables. Given the exploratory nature of this analysis, a minimum Z-score threshold of 1.3 was applied to detect significant clusters of cortical variation, reduce the likelihood of including random fluctuations, and increase sensitivity. This relatively liberal vertex-wise threshold increases the likelihood of detecting true effects in a small sample but also increases susceptibility to false positives. To address this, after clusters were identified, statistical significance was assessed for each cluster by applying a stringent cluster-wise correction (CWP ≤ 0.05), with the resulting CWP representing the multiple-comparison-corrected p-value. 

Cluster-wise correction was performed separately for each analysis: left hemisphere thickness, right hemisphere thickness, left hemisphere volume, and right hemisphere volume, resulting in four independent multiple-comparison corrections. The CWP represents the multiple-comparison-corrected p-value for each identified cluster. When multiple significant clusters were identified within the same anatomical region, each represents a spatially distinct area of structural variation with unique coordinates and statistical parameters, as determined by FreeSurfer's automated vertex-wise parcellation.

Results were presented as maximum Z-scores, corresponding cluster-wise p-values, and anatomical locations with MNI coordinates.

## Results

FreeSurfer analysis revealed significant alterations in cortical thickness and volume across the mixed-age ADHD cohort compared to pediatric controls (Tables 1-6). In some cases, multiple significant clusters were identified within the same anatomical region, reflecting spatially distinct areas of structural variation. In the right hemisphere, reduced cortical thickness was observed in the superior frontal gyrus (max Z = -3.383, size = 155.08 mm², MNIxyz: 14.9, 5.8, 61.0, CWP = 0.0002). The right hemisphere also showed several clusters with significant increases or decreases in volume relative to the control. Clusters with the most substantial evidence demonstrated a CWP ≤ 0.0002 and included an increased volume observed in the precentral gyrus (max Z = 5.357, size = 79.58 mm², MNIxyz: 48.8, 4.3, 16.3), postcentral gyrus (max Z = 5.4344, size = 64.86 mm², MNIxyz: 46.7, -23.7, 44.2), lingual gyrus (max Z = 4.0801, size = 37.27 mm², MNIxyz: 24.4, -61.0, -6.3), and fusiform gyrus (Max Z = 4.5189, Size = 28.06 mm², MNIxyz: 36.4, -12.5, -32.9), while decreased volume was observed in the superior frontal gyrus (max Z = -3.5884, size = 27.42 mm², MNIxyz: 10.3, 16.1, 62.2). Bidirectional volume changes (both increases and decreases) were found in the lateral occipital gyrus (max Z = 4.4186, size = 31.05 mm², MNIxyz: 14.5, -92.0, 15.8; max Z = -4.0215, size = 47.93 mm², MNIxyz: 45.2, -74.6, -11.7) and the rostral middle frontal cortex (max Z = 3.9289, size = 26.82 mm², MNIxyz: 38.2, 19.0, 28.5; max Z = -2.9637, size = 31.48 mm², MNIxyz: 22.4, 59.2, 14.1). 

**Table 1:** Cortical thickness: morphometric characteristics ^a^Surface area of the cluster in square millimeters. ^b^Montreal Neurological Institute coordinates of cluster center (X, Y, Z axes) demonstrating the spatial location of the cluster.

Hemisphere	Location	Size (mm^2^)^a^	MNIX^b^	MNIY^b^	MNIZ^b^
Right	Superior frontal	155.08	14.9	5.8	61
Left	Postcentral	98.69	-59.2	-15.1	29.9

**Table 2:** Cortical thickness: statistical parameters ^a^Maximum Z-score (vertex-wise general linear model (GLM) test statistic converted from t-statistics). ^b^Vertex number at the maximum Z-score. ^c^Cluster-wise corrected p-values (CWP).

Hemisphere	Location	Max Z (test-statistic)^a^	VtxMax^b^	Cluster-wise corrected p-values (CWP)^c^
Right	Superior frontal	-3.383	58090	0.0002
Left	Postcentral	4.0145	81049	0.00479

**Table 3:** Right hemisphere volume: morphometric characteristics ^a^Surface area of the cluster in square millimeters. ^b^Montreal Neurological Institute coordinates of cluster center (X, Y, Z axes) demonstrating the spatial location of the cluster. Note: Multiple entries for the same anatomical region reflect distinct clusters identified within that region.

Location	Size (mm^2^)^a^	MNIX^b^	MNIY^b^	MNIZ^b^
Precentral	79.58	48.8	4.3	16.3
Postcentral	64.86	46.7	-23.7	44.2
Lateral occipital	47.93	45.2	-74.6	-11.7
Lingual	37.27	24.4	-61	-6.3
Rostral middle frontal	31.48	22.4	59.2	14.1
Lateral occipital	31.05	14.5	-92	15.8
Fusiform	28.06	36.4	-12.5	-32.9
Superior frontal	27.42	10.3	16.1	62.2
Rostral middle frontal	26.82	38.2	19	28.5
Rostral middle frontal	25.36	38.4	32.8	18.2
Pars triangularis	24.36	51.6	26.3	4.7
Cuneus	23.31	6.5	-89	15.1
Postcentral	20.85	48.3	6.6	-31.3
Middle temporal	28.06	36.4	-12.5	-32.9
Rostral middle frontal	20.15	47.6	29.2	11.1
Pars opercularis	19.31	52.8	19.9	13.7
Inferior temporal	18.07	44.2	-5	-41.7
Lateral orbitofrontal	16.76	28.5	22.3	-4.3
Lateral orbitofrontal	16.64	16.8	37.8	-21.3

**Table 4:** Right hemisphere volume: statistical parameters ^a^Maximum Z-score (vertex-wise general linear model (GLM) test statistic converted from t-statistics). ^b^Vertex number at the maximum Z-score. ^c^Cluster-wise corrected p-values (CWP). Note: Multiple entries for the same anatomical region reflect distinct clusters identified within that region.

Location	Max Z (test-statistic)^a^	VtxMax^b^	Cluster-wise corrected p-values (CWP)^c^
Precentral	5.357	94098	0.0002
Postcentral	5.4344	26775	0.0002
Lateral occipital	-4.0215	143960	0.0002
Lingual	4.0801	143753	0.0002
Rostral middle frontal	-2.9637	106364	0.0002
Lateral occipital	4.4186	101262	0.0002
Fusiform	4.5189	24356	0.0002
Superior frontal	-3.5884	92254	0.0002
Rostral middle frontal	3.9289	19848	0.0002
Rostral middle frontal	3.9407	46677	0.0006
Pars triangularis	-3.4527	106088	0.0008
Cuneus	4.7836	21044	0.0014
Postcentral	4.5623	115907	0.0036
Middle temporal	4.5189	24356	0.0002
Rostral middle frontal	-2.6441	131368	0.00559
Pars opercularis	6.6337	109872	0.00838
Inferior temporal	5.8872	101736	0.01613
Lateral orbitofrontal	-7.3406	110347	0.03214
Lateral orbitofrontal	3.2582	55220	0.0343

**Table 5:** Left hemisphere volume: morphometric characteristics ^a^Surface area of the cluster in square millimeters. ^b^Montreal Neurological Institute coordinates of cluster center (X, Y, Z axes) demonstrating the spatial location of the cluster. Note: Multiple entries for the same anatomical region reflect distinct clusters identified within that region.

Location	Size (mm^2^)^a^	MNIX^b^	MNIY^b^	MNIZ^b^
Postcentral	127.71	-42.2	-34.2	53.8
Lateral occipital	56.45	-33.1	-80.5	5
Supramarginal	40.92	-37.4	-41.1	37.9
Inferior parietal	29.7	-45.5	-72.1	10.1
Rostral middle frontal	29.38	-22.6	47.9	28.3
Superior frontal	26.88	-11.1	6.3	40.2
Inferior parietal	24.44	-43.2	-60.4	12.1
Lateral occipital	23.89	-39.5	-69.4	-9
Supramarginal	22.52	-48.9	-54.6	29.4
Lingual	21.44	-21.1	-71.3	-7.1
Postcentral	21.1	-26.4	-34.4	62.8
Caudal middle frontal	20.77	-26.8	14.2	40.8
Inferior temporal	20.17	-47.7	-62.9	-5.8
Caudal middle frontal	18.92	-33.5	9	55.1
Inferior temporal	18.92	-49.2	-48.7	-16
Postcentral	17.95	-48.4	-17.3	15
Rostral middle frontal	17.57	-31.3	46.3	15.6

**Table 6:** Left hemisphere volume: statistical parameters ^a^Maximum Z-score (vertex-wise general linear model (GLM) test statistic converted from t-statistics). ^b^Vertex number at the maximum Z-score. ^c^Cluster-wise corrected p-values (CWP). Note: Multiple entries for the same anatomical region reflect distinct clusters identified within that region.

Location	Max Z (test-statistic)^a^	VtxMax^b^	Cluster-wise corrected p-values (CWP)^c^
Postcentral	8.3353	16460	0.0002
Lateral occipital	4.515	87267	0.0002
Supramarginal	-4.2808	59884	0.0002
Inferior parietal	4.2486	51760	0.0002
Rostral middle frontal	5.0197	63485	0.0002
Superior frontal	-4.3236	124643	0.0004
Inferior parietal	4.9689	156938	0.0008
Lateral occipital	-4.9325	161849	0.001
Supramarginal	-4.1639	106750	0.0016
Lingual	4.2918	102062	0.0028
Postcentral	4.3521	59788	0.003
Caudal middle frontal	-3.831	103553	0.003
Inferior temporal	3.9481	34205	0.004
Caudal middle frontal	-3.8733	23139	0.00918
Inferior temporal	4.6115	70065	0.00918
Postcentral	-3.6702	65802	0.01554
Rostral middle frontal	3.5075	7282	0.01851

Volume cluster changes with moderate evidence demonstrated a CWP ≤ 0.05, but >0.0002, and included decreased volume in the pars triangularis (max Z = -3.4527, size = 24.36 mm², MNIxyz: 51.6, 26.3, 4.7; max Z = -2.6441, size = 20.15 mm², MNIxyz: 47.6, 29.2, 11.1), and increased volume in the cuneus (max Z = 4.7836, size = 23.31 mm², MNIxyz: 6.5, -89.0, 15.1), middle temporal gyrus (max Z = 4.5623, size = 20.85 mm², MNIxyz: 48.3, 6.6, -31.3), pars opercularis (max Z = 6.6337, size = 19.31 mm², MNIxyz: 52.8, 19.9, 13.7), and inferior temporal gyrus (max Z = 5.8872, size = 18.07 mm², MNIxyz: 44.2, -5.0, -41.7), as well as bidirectional changes in the lateral orbitofrontal gyrus (max Z = -7.3406, size = 16.76 mm², MNIxyz: 28.5, 22.3, -4.3; max Z = 3.2582, size = 16.64 mm², MNIxyz: 16.8, 37.8, -21.3). 

In the left hemisphere, increased thickness was observed in the postcentral gyrus (max Z = 4.0145, size = 98.69 mm², MNIxyz: -59.2, -15.1, 29.9, CWP = 0.00479). Volume change clusters with the most substantial evidence demonstrated a CWP ≤ 0.0002 and included an increased volume observed in the postcentral gyrus (max Z = 8.3353, size = 127.71 mm², MNIxyz: -42.2, -34.2, 53.8), lateral occipital gyrus (max Z = 4.515, size = 56.45 mm², MNIxyz: -33.1, -80.5, 5.0), (max Z = 4.2486, size = 29.7 mm², MNIxyz: -45.5, -72.1, 10.1), and rostral middle frontal cortex (max Z = 5.0197, size = 29.38 mm², MNIxyz: -22.6, 47.9, 28.3), and decreased volume in the supramarginal gyrus (max Z = -4.2808, size = 40.92 mm², MNIxyz: -37.4, -41.1, 37.9).

**Figure 2:**
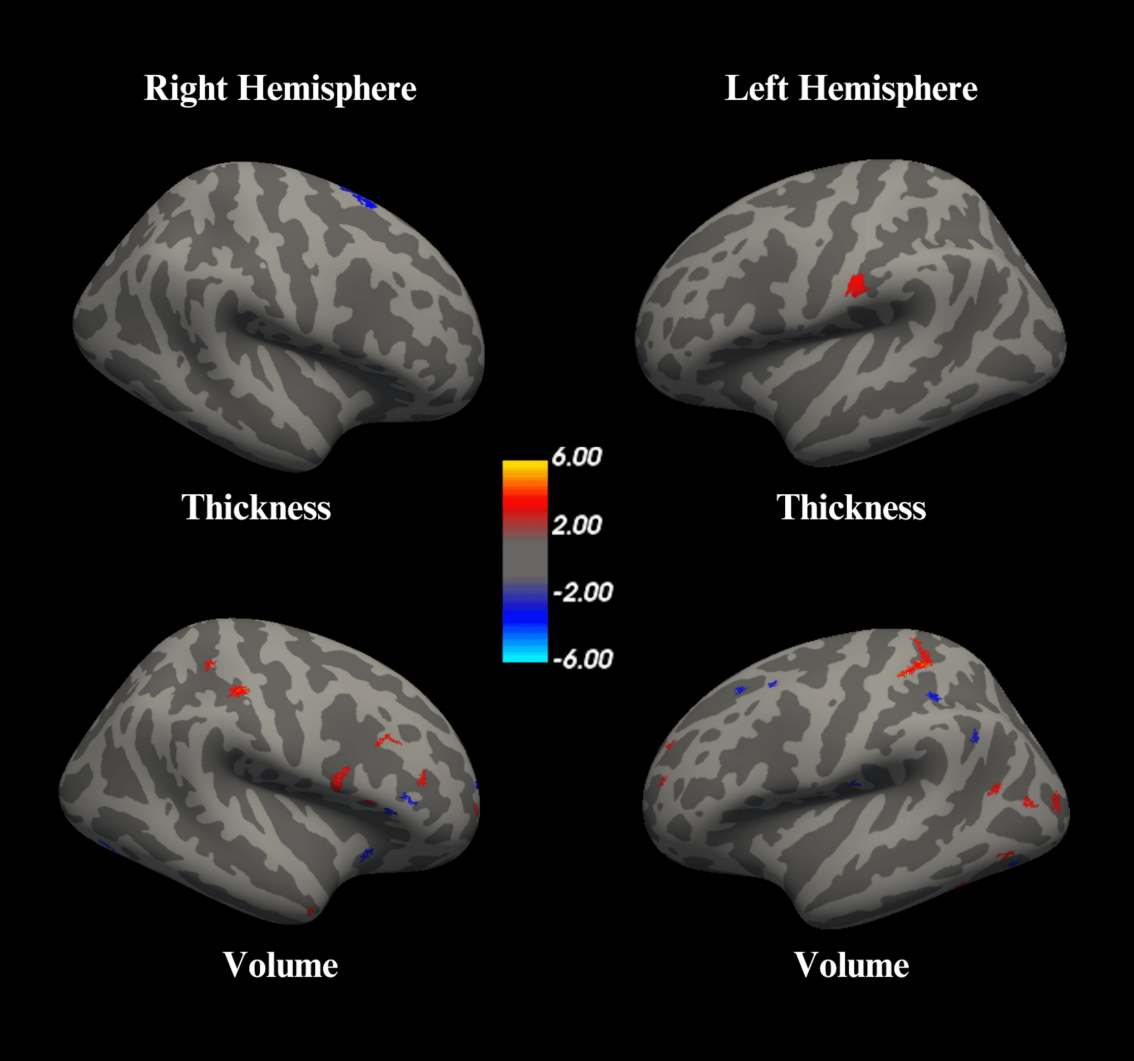
Surface-based morphometry (SBM) maps showing significant differences in cortical thickness and volume Red-yellow = increases; blue = decreases. Threshold: Z ≥ ±1.3, CWP ≤ 0.05.

Volume cluster changes with moderate evidence demonstrated a CWP ≤ 0.05, but >0.0002, and included decreased volume in the superior frontal cortex (max Z = -4.3236, size = 26.88 mm², MNIxyz: -11.1, 6.3, 40.2), lateral occipital cortex (max Z = -4.9325, size = 23.89 mm², MNIxyz: -39.5, -69.4, -9.0), supramarginal gyrus (max Z = -4.1639, size = 22.52 mm², MNIxyz: -48.9, -54.6, 29.4), caudal middle frontal cortex (max Z = -3.831, size = 20.77 mm², MNIxyz: -26.8, 14.2, 40.8; max Z = -3.8733, size = 18.92 mm², MNIxyz: -33.5, 9.0, 55.1), and increased volume in the inferior parietal cortex (max Z = 4.9689, size = 24.44 mm², MNIxyz: -43.2, -60.4, 12.1), lingual gyrus (max Z = 4.2918, size = 21.44 mm², MNIxyz: -21.1, -71.3, -7.1), and inferior temporal cortex (max Z = 3.9481, size = 20.17 mm², MNIxyz: -47.7, -62.9, -5.8; max Z = 4.6115, size = 18.92 mm², MNIxyz: -49.2, -48.7, -16.0). The postcentral gyrus also demonstrated bidirectionality with decreased volume (max Z = -3.6702, size = 17.95 mm², MNI: -48.4, -17.3, 15.0, CWP = 0.01554). The cortical regions exhibiting significant thickness and volume alterations are visualized in Figure 2. 

## Discussion

Key results

This study aimed to investigate structural brain alterations in ADHD using SBM by using a publicly available dataset from OpenNeuro.org. Our findings revealed significant variations in cortical thickness and volume between individuals with ADHD and neurotypical controls, primarily within regions implicated in executive function, sensory processing, and motor control. Specifically, we observed reduced cortical thickness in the right superior frontal gyrus and both increased and decreased volumes in various regions, including the precentral, postcentral, lingual, fusiform, lateral occipital, and rostral middle frontal gyri. In the left hemisphere, increased cortical thickness was found in the postcentral gyrus, along with volume alterations in the postcentral, lateral occipital, inferior parietal, supramarginal, and rostral middle frontal regions.

Interpretation

These results align with previous neuroimaging studies that have reported structural differences in ADHD, particularly in prefrontal and parietal areas [6,9-14]. The observed reductions in cortical thickness and alterations in gray matter volume may reflect underlying neuropathological processes contributing to the cognitive and behavioral symptoms associated with ADHD. For example, changes in the prefrontal cortex, a region critical for executive functions, such as attention and working memory, may explain the inattentive and impulsive behaviors often seen in individuals with ADHD. These results expand prior work by showing that cortical alterations are not uniformly reduced but exhibit both increases and decreases in volume, consistent with the possibility of region-specific compensatory or maturational mechanisms rather than global cortical thinning. This pattern is consistent with emerging models of ADHD as a disorder of atypical brain maturation, where delayed development in prefrontal and parietal cortices coexists with regionally accelerated or compensatory growth in sensorimotor areas [5,10,14]. However, given the developmental heterogeneity of the sample, these findings may reflect a combination of ADHD-related and age-related structural differences, despite statistical adjustment for age. Notably, the ADHD group in this study included both adult and pediatric participants, whereas the control group consisted exclusively of pediatric individuals. This imbalance may contribute to the observed bidirectional structural findings. Prior work has demonstrated that ADHD is associated with delayed cortical maturation, as well as alterations in synaptic pruning processes, which may manifest as either cortical thinning or relative thickening depending on developmental stage [17,20,21]. In this context, the observed increases and decreases in cortical measures may reflect a combination of disorder-related and age-related neurodevelopmental effects rather than ADHD-specific changes alone. These interpretations remain hypothesis-generating and require validation through longitudinal, age-matched studies.

Furthermore, by applying SBM, this study adds precision to previous voxel-based findings, allowing for vertex-level localization of cortical changes with greater anatomical specificity. This methodological approach contributes to the growing effort to refine structural biomarkers of ADHD by mapping subtle, spatially distinct cortical variations that may underlie the heterogeneity of clinical symptoms. A significant aspect of this study is its reliance on a publicly accessible dataset, demonstrating the feasibility and potential of open data resources in neuroimaging research. This approach serves as a proof-of-concept, showing how valuable insights can be derived from existing datasets. By utilizing the “Response inhibition and selective attention in adults and children with and without ADHD” dataset from OpenNeuro.org, we were able to conduct a comprehensive SBM analysis with a small sample size, highlighting the efficiency of leveraging open data to advance the field.

Limitations

Several limitations must be acknowledged. First, and most critically, the analytic sample included participants across a range of ages, introducing developmental heterogeneity. Specifically, the ADHD group included both adult and pediatric participants, whereas the control group consisted exclusively of pediatric individuals. While we included age as a continuous covariate in our statistical model and applied cluster-wise correction to account for developmental variability, this age imbalance represents a limitation that may not be fully addressed through covariate adjustment alone. Nonlinear or region-specific developmental effects may not be completely captured by this approach, limiting our ability to definitively isolate ADHD-related alterations from age-related neurodevelopmental differences. Consequently, these findings should not be interpreted as ADHD-specific biomarkers, and age-matched replication is essential. Second, the sample size of the ADHD group was relatively small, limiting statistical power and generalizability of our findings. Third, information on medication status, including stimulant use, was not available in the dataset. Given that stimulant exposure has been associated with structural brain changes, this represents a potential confounding factor. Moreover, other potentially important clinical variables were unavailable, including ADHD subtype (inattentive, hyperactive-impulsive, combined), comorbid psychiatric conditions, and symptom severity, limiting our ability to characterize clinical heterogeneity within the ADHD group. Fourth, several statistical considerations limit interpretation. The use of a relatively liberal vertex-wise threshold (Z = 1.3) prioritized sensitivity in this exploratory analysis but increases susceptibility to false-positive findings despite cluster-wise correction. Additionally, our statistical model included age as a linear covariate but did not test for age-by-group interactions, meaning we assumed parallel age-related effects across ADHD and control groups. Given the known non-linear trajectory of cortical development and evidence for delayed maturation in ADHD, the absence of interaction terms may have limited our ability to detect group differences that vary by developmental stage. Fifth, the cross-sectional design precludes any causal inferences regarding the relationship between brain structure and ADHD. Longitudinal studies are needed to determine whether observed structural variations represent primary ADHD-related alterations, secondary compensatory changes, consequences of chronic symptom burden, or treatment effects. Finally, our analysis was limited to structural MRI data and did not incorporate functional connectivity, task-based activation, or other multimodal neuroimaging measures. Future studies should incorporate larger, age-stratified cohorts with comprehensive clinical characterization and multimodal neuroimaging to better isolate ADHD-specific structural alterations and validate these exploratory findings.

Generalizability

Despite these limitations, this study provides valuable insights into the structural brain alterations associated with ADHD and highlights the utility of open datasets in neuroimaging research. Future research should aim to validate these findings in larger, more diverse samples and explore the longitudinal changes in brain structure associated with ADHD. Additionally, further studies could explore the functional implications of these structural differences, potentially using the functional MRI data also available in the OpenNeuro.org dataset. Overall, this study underscores the potential of open data to advance our understanding of neurodevelopmental disorders like ADHD and encourages the broader adoption of data-sharing practices in the neuroimaging community.

## Conclusions

Using SBM and statistical corrections, we identified region-specific structural variations in cortical thickness and volume associated with ADHD across a developmentally heterogeneous sample. Rather than following a uniform pattern, the alterations we observed point to a more nuanced neurodevelopmental profile, one that involves multiple networks responsible for attention, executive function, sensory processing, and visual integration. 

Our results support a multi-network understanding of ADHD and highlight the need to look beyond prefrontal circuits and consider how interactions between sensory, visual, and executive systems might shape both symptoms and the developmental trajectory of the disorder. Future studies should continue exploring how these brain differences map onto individual experiences, treatment outcomes, and functional abilities across the lifespan. Equally important is how we reached these findings. By leveraging an open-access neuroimaging dataset, we demonstrated that meaningful structural insights can be derived even from relatively small samples. This emphasized the value of publicly available resources in advancing neuroscience research, not just by improving access, but by enhancing reproducibility, transparency, and collaboration across the research community. 
